# Inhibition of Jak/STAT signaling reduces the activation of pancreatic stellate cells *in vitro* and limits caerulein-induced chronic pancreatitis *in vivo*

**DOI:** 10.1038/s41598-017-01973-0

**Published:** 2017-05-11

**Authors:** Hannah M. Komar, Gregory Serpa, Claire Kerscher, Erin Schwoegl, Thomas A. Mace, Ming Jin, Ming-Chen Yang, Ching-Shih Chen, Mark Bloomston, Michael C. Ostrowski, Phil A. Hart, Darwin L. Conwell, Gregory B. Lesinski

**Affiliations:** 1 Comprehensive Cancer Center, The Arthur G. James Cancer Hospital and Richard J. Solove Research Institute, The Ohio State University, Columbus, OH USA; 2 The Ohio State University, Columbus, OH USA; 3 Division of Gastroenterology, Hepatology, and Nutrition, The Ohio State University Wexner Medical Center, Columbus, OH USA; 4 Department of Pathology, The Ohio State University Wexner Medical Center, Columbus, OH USA; 5 College of Pharmacy, The Ohio State University, Columbus, OH USA; 6 South Florida Surgical Oncology, Fort Myers, FL USA; 7 Department of Cancer Biology and Genetics, The Ohio State University, Columbus, OH USA; 8 Department of Hematology and Oncology, Winship Cancer Institute of Emory University, Atlanta, GA USA

## Abstract

Chronic pancreatitis (CP) is a fibro-inflammatory disease leading to pain, maldigestion, and pancreatic insufficiency. No therapeutic options exist due to a limited understanding of the biology of CP pathology. Recent findings implicate pancreatic stellate cells (PSC) as prominent mediators of inflammatory and fibrotic processes during CP. Here, we utilized primary and immortalized PSC obtained from mice and patients with CP or pancreatic cancer to examine the effect of Jak/STAT and MAPK pathway inhibition *in vitro*. The well-characterized caerulein model of CP was used to assess the therapeutic efficacy of Jak1/2 inhibition *in vivo*. Treatment of cultured PSC with the Jak1/2 inhibitor ruxolitinib reduced STAT3 phosphorylation, cell proliferation, and expression of alpha-smooth muscle actin (α-SMA), a marker of PSC activation. Treatment with the MAPK inhibitor, MEK162, had less consistent effects on PSC proliferation and no impact on activation. In the caerulein-induced murine model of CP, administration of ruxolitinib for one week significantly reduced biomarkers of inflammation and fibrosis. These data suggest that the Jak/STAT pathway plays a prominent role in PSC proliferation and activation. *In vivo* treatment with the Jak1/2 inhibitor ruxolitinib reduced the severity of experimental CP, suggesting that targeting Jak/STAT signaling may represent a promising therapeutic strategy for CP.

## Introduction

Chronic pancreatitis (CP) is characterized by persistent inflammation and fibrosis of the pancreas. Patients with CP often experience recurrent abdominal pain, nausea, and maldigestion that progress to exocrine insufficiency, fat-soluble vitamin deficiency, metabolic bone disease, and diabetes mellitus^1–6^. Depending on etiology, CP patients also have an approximate 3–5-fold increased risk of developing pancreas cancer^7–12^. To date, no clinical therapy is available to reverse the inflammatory damage associated with CP. Instead, management is focused on treatment of associated symptoms and complications. Thus, identifying novel interventions for this disease represents a high priority and would fill an unmet medical need to improve quality of life, reduce risk of malignancy, and limit medical costs associated with long-term care of these patients^13, 14^.

The fibro-inflammatory response associated with CP is hypothesized to result from premature activation of pancreatic enzymes within the gland, resulting in auto-digestion of parenchyma. Subsequent acute inflammatory events result in release of pro-inflammatory mediators that promote both immune cell infiltration and activation of local fibroblasts termed pancreatic stellate cells (PSC). Once active, PSC promote inflammation and fibrosis through secretion of cytokines and chemokines as well as matrix metalloproteinases (MMPs), tissue inhibitor of metalloproteinases (TIMPs), and collagen^15–17^. Transient PSC activation occurs during instances of acute pancreatic inflammation, however the onset of CP is characterized by PSC that display a constitutively active phenotype to promote a state of chronic fibro-inflammation^18, 19^. The proportion of patients with acute pancreatitis (AP) that will progress to CP varies by etiology. Specifically, the development of CP is more common among those with acute alcoholic pancreatitis compared to acute gallstone pancreatitis. This difference may be due, in part, to the reduced viability of PSC following exposure to bile acids during acute gallstone pancreatitis^20^. This suggests the importance of PSC activity to the transition from acute inflammation to CP. Activated PSC are also observed in pancreatic ductal adenocarcinoma (PDAC), where they support growth and invasiveness of tumors^21–23^. Accordingly, PSC may represent a viable therapeutic target in the context of CP to reduce inflammation, fibrosis, and risk of malignancy.

PSC secrete high levels of several immunomodulatory factors, including interleukin-6 (IL-6), tumor necrosis factor alpha (TNFα), monocyte chemoattractant protein-1 (MCP-1), and others^24^. Many of these factors act in an autocrine and paracrine manner to orchestrate continued PSC survival, cellular activation, and proliferation while driving fibro-inflammatory processes that contribute to CP pathology^25–28^. IL-6 and other cytokines exert their effects via the transmembrane receptor gp130 to activate Jak/STAT signaling, notably Jak2/STAT3. Once activated, STAT3 positively regulates several pro-survival and pro-inflammatory gene signatures. The Jak/STAT pathway also cross-talks with other signal transduction pathways including MAPK and NF-kB to amplify expression of inflammatory genes^29–32^. Data from animal models and human patients suggest that IL-6 signaling is of particular importance in the context of CP. In murine models of disease, genetic ablation of IL-6 reduces susceptibility to caerulein-induced pancreatitis and associated lung injury^33^. Serum levels of IL-6 are also often found to be elevated in human CP patients^34–37^. Although acquisition of human pancreatic tissue across the spectrum of CP disease stages is not feasible, several studies have explored the role of this pathway in the context of PDAC. IHC analysis of human PDAC tumors revealed robust staining of IL-6 localized to the stromal compartment, which includes PSC, immune cells, and others^38^. Furthermore, murine models of PDAC have demonstrated cooperation between STAT3 signaling and activated *KRas* within the pancreas to drive cancer progression^39^. Thus, stromal-derived IL-6/Jak2/STAT3 signaling appears to play a prominent role in PSC activity, CP pathology, and PDAC development.

To our knowledge there are currently no clinical trials and only limited *in vitro* or *in vivo* studies targeting soluble IL-6 or the Jak/STAT pathway in the context of CP^40^. Although development of clinically suitable STAT3 inhibitors is lacking, considerable advances have been made in the development of small molecule inhibitors of the upstream Jak proteins^41, 42^. These agents are well tolerated by patients and are FDA-approved for treatment of other inflammatory disorders including rheumatoid arthritis, myelofibrosis and polycythemia vera^43–45^. However, Jak inhibitors have never been formally tested in patients with CP.

We sought to characterize the activation of pro-inflammatory STAT3 and MAPK pathways in PSC from both CP and PDAC, and to assess the ability of targeted inhibition to limit pathologic PSC activity. We hypothesized that inhibition of Jak/STAT or MAPK signaling would reduce PSC activity and limit the severity of caerulein-induced CP. Our results demonstrate that both the STAT3 and MAPK pathways are activated in cultured mouse and human PSC from the setting of CP and PDAC. Inhibition of Jak1/2 resulted in decreased proliferation of PSC, which was associated with diminished cellular activation. The impact of MEK inhibition was more variable, depending on the cell culture assayed. In a proof-of-concept study using the caerulein-induced murine model of CP, short-term treatment with ruxolitinib, a Jak1/2 inhibitor, led to partial resoration of serum lipase levels and reduced acinar cell loss and fibrosis. These findings suggest that Jak/STAT inhibition may limit the pathology observed in caerulein-induced pancreatitis.

## Results

### α-SMA^+^ PSC display STAT3 and MAPK signaling and secrete immunomodulatory factors

**Figure 1:**
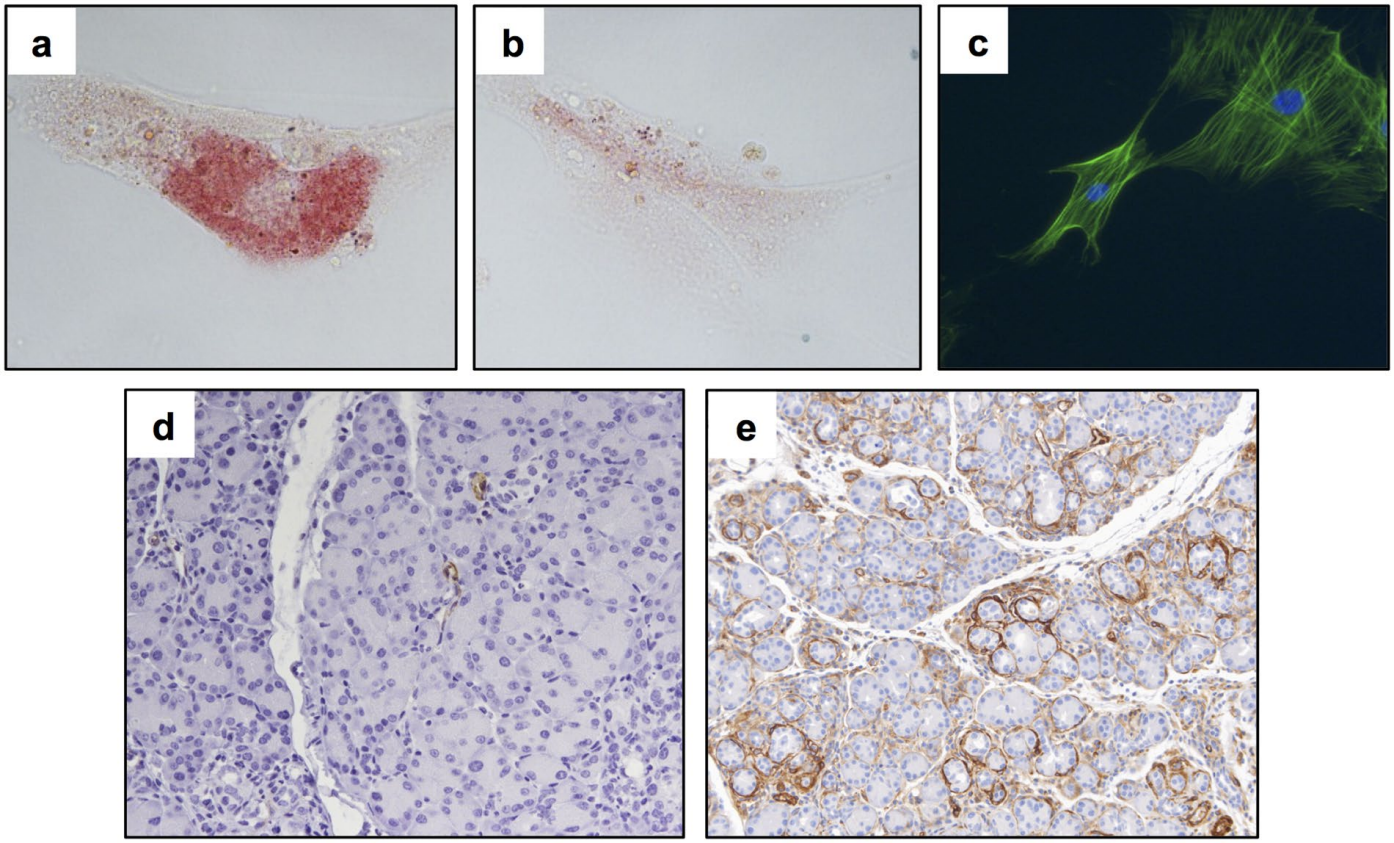
PSC display an activated phenotype in culture and in a murine model of CP. PaSC were treated with **(a)** 10 μM all-trans retinoic acid (ATRA) or **(b)** vehicle control for 48 hours and stained for Oil-Red O. Cells were analyzed by light microscopy at 40X magnification. **(c)** Untreated PaSC were stained for α-SMA (green) by fluorescent microscopy following 48 hours of incubation (DAPI counterstain, 40X magnification). **(d)** Formalin fixed paraffin embedded (FFPE) pancreatic tissue from mice with caerulein-induced pancreatitis after **(d)** 1 week and **(e)** 5 weeks of treatment were stained for α-SMA (20X magnification). Representative images from n = 5 mice per group.

**Figure 2:**
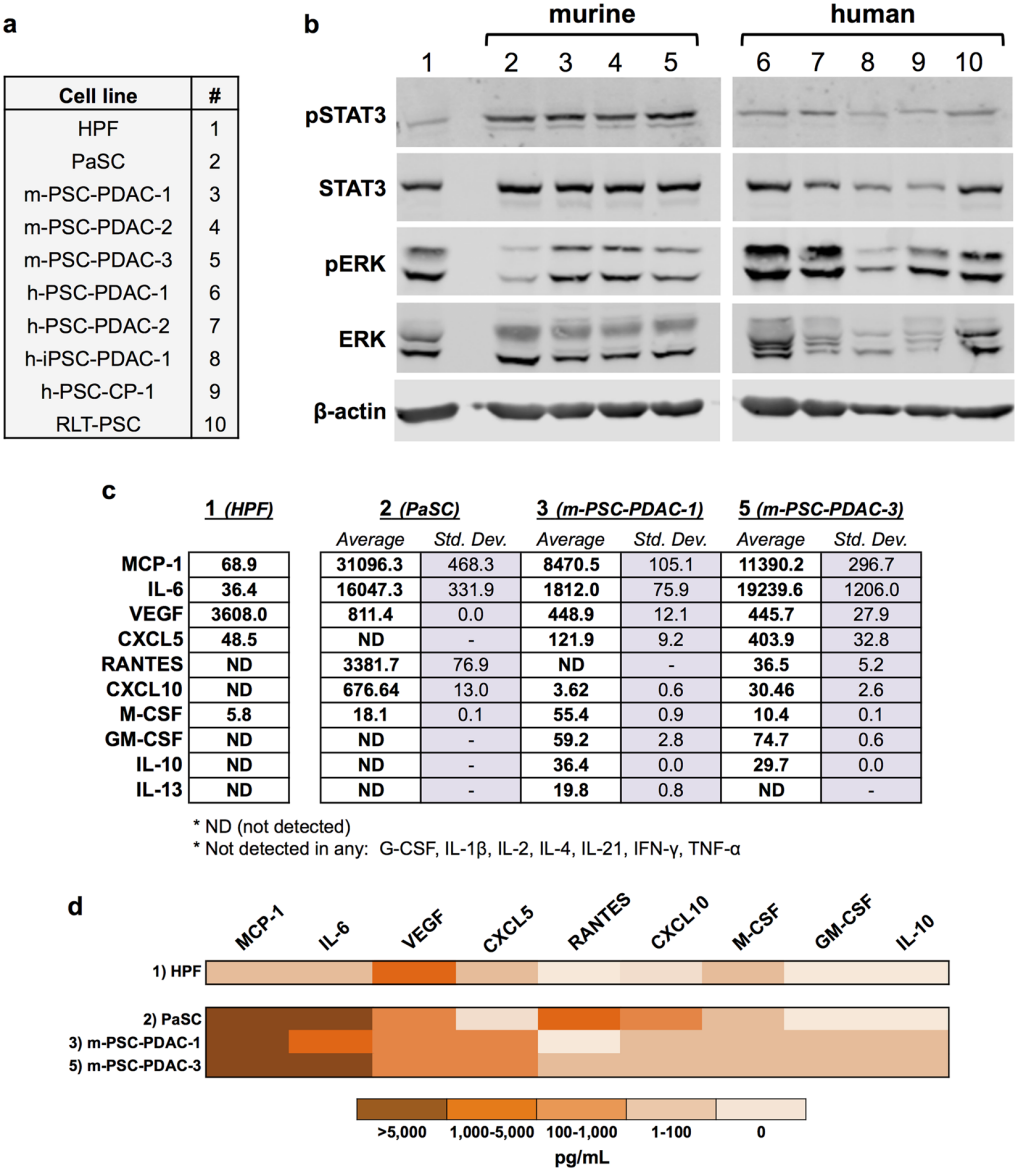
PSC display constitutive activation of STAT3 and MAPK signaling and secrete pro-inflammatory factors. (**a**) Summary of all PSC and PSC cell cultures used. **(b)** Cells were grown in DMEM and lysed once they reached 70–80% confluence for western blot analysis. pSTAT3, STAT3, pERK, and ERK were analyzed by immunoblot. β-actin served as a loading control. **(c)** Supernatants from 3 murine PSC cell lines and the human control pancreas-derived fibroblast line (HFP) (detailed in Table 1) were collected from 70% confluent cells grown in DMEM. A panel of cytokines and chemokines was analyzed in these supernatants by Luminex assay. Values are listed as an average of two replicates in pg/mL with the standard deviation. ND indicates analytes were assayed, but the levels were detected beneath the lowest standard curve reference value. **(d)** Selected results from the Luminex assay, presented as a heat map.

PSC lines (Table 1) were purchased or PSC cell cultures were isolated from mouse or human pancreatic tissue as described and were characterized^24^. In culture, PSC exhibited a pseudoquiescent phenotype when incubated with 10 μM all-trans retinoic acid (ATRA), as evidenced by intracellular Oil-Red O positive lipid droplets **(**Fig. 1a **)**. Under normal culture conditions, PSC typically assume an activated state, and are thus negative for Oil-Red O staining, while positive for α-SMA, a marker of stellate cell activation **(**Fig. 1b,c **)**. These active PSC were evident in pancreata from the caerulein-induced murine model of CP and increased with disease severity **(**Fig. 1d,e **)**. Lysates prepared from activated PSC grown to 70–80% confluence demonstrated constitutive phosphorylation of STAT3 and ERK proteins in all cell lines tested, indicating activation of the pro-inflammatory, pro-survival STAT3 and MAPK pathways **(**Fig. 2a,b **)**. Supernatants from these cells further demonstrated elevated levels of several immunomodulatory factors, including IL-6, MCP-1, and CXCL10, as compared to a human pancreas-derived fibroblast line (HPF), which served as a control (Fig. 2c,d).

**Table 1:** Human and murine pancreatic stellate cell lines utilized *in vitro*. Details, including cell type, species of origin, immortalization status, and disease of origin, are provided for each cell line (PDAC indicates *pancreatic ductal adenocarcinoma*, GEMM indicates *genetically engineered mouse model*).

#	Name	Cell Type	Species	Immortalization Status	Disease of origin/Genotype
1	HPF	fetal pancreatic fibroblast	human	non-immortalized	Normal
2	PaSC	PSC	mouse	immortalized	Normal
3	m-PSC-PDAC-1	PSC	mouse	non-immortalized	PDAC GEMM: *Mist1(KRAS* ^*G12D*/+^)
4	m-PSC-PDAC-2	PSC	mouse	non-immortalized	PDAC GEMM: *Mist1*(*KRAS* ^*G12D*/+^)
5	m-PSC-PDAC-3	PSC	mouse	non-immortalized	PDAC GEMM: (*Brca1* ^*flox2*/*flox2*^; *Kras* ^*LSL*-*G12D*/+^; *p53* ^*LSL*-*R27OH*/+^; *Pdx1-cre*)
6	h-PSC-PDAC-1	PSC	human	non-immortalized	PDAC
7	h-PSC-PDAC-2	PSC	human	non-immortalized	PDAC
8	h-iPSC-PDAC-1	PSC	human	immortalized	PDAC
9	h-PSC-CP-1	PSC	human	non-immortalized	chronic pancreatitis
10	RLT-PSC	PSC	human	immortalized	chronic pancreatitis

### Effects of the Jak1/2 inhibitor, ruxolitinib, and the MEK inhibitor, MEK162, on proliferation of PSC *in vitro*

**Figure 3:**
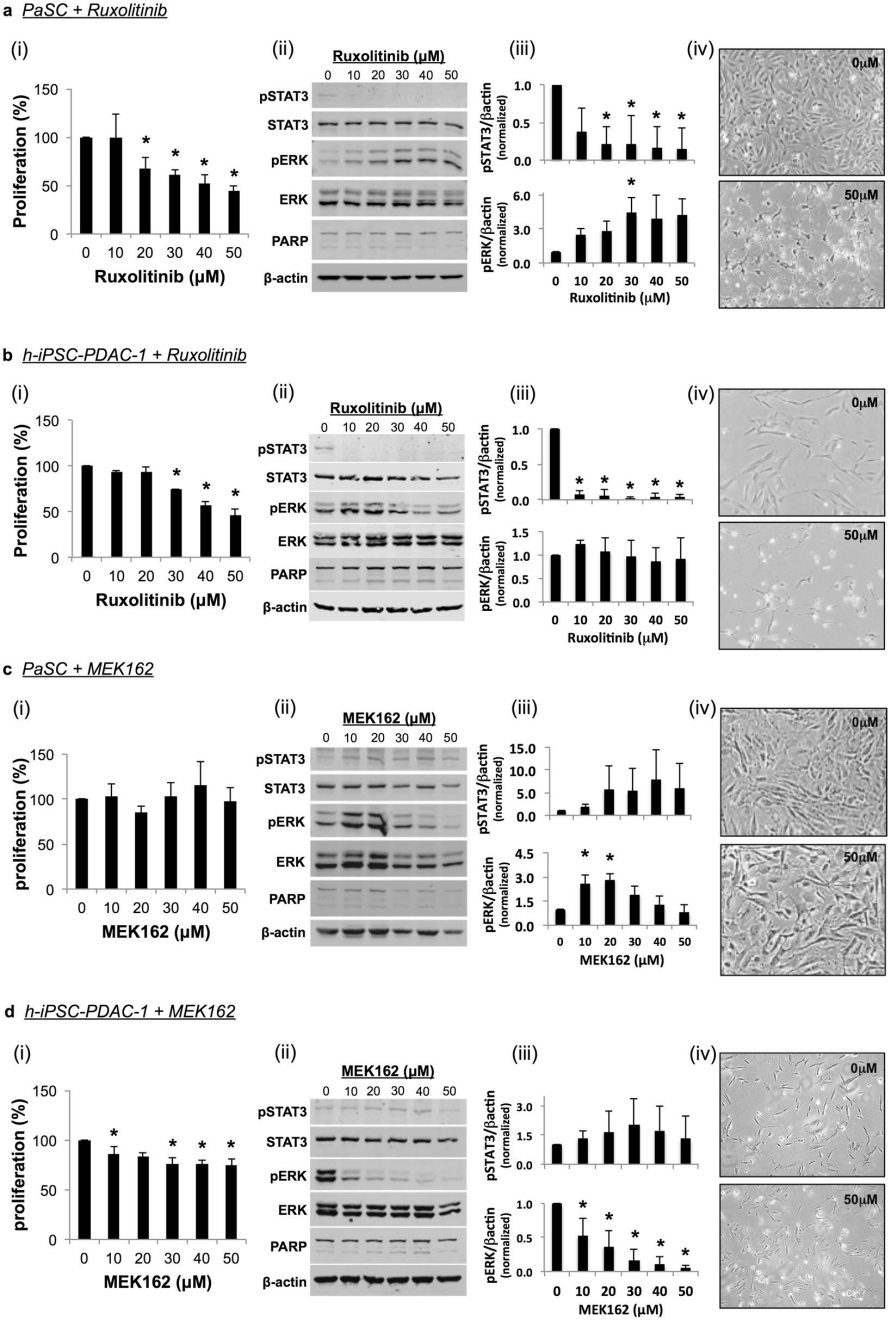
Effect of Jak/STAT and MEK inhibition on PSC proliferation *in vitro*. **(a,c**) PaSC or **(b,d)** h-iPSC-PDAC-1 were treated with ruxolitinib **(a,b)** or MEK162 **(c,d)**. **(i)** After 24 hours of incubation, cell proliferation was analyzed by MTT assay. **(ii)** Lysates were also taken at 24 hours and analyzed by western blot. β-actin served as a loading control. **(iii)** Results were quantified via densitometry and normalized to β-actin. Graphs display mean ± STD from 3 biological replicates (* indicates p < 0.05). (**iv**) Light microscopy images were taken of treated cells following 72 hours of incubation (40X magnification).

Because the inflammatory, pro-survival Jak-STAT and MAPK pathways were activated in PSC, we investigated the effects of inhibiting these pathways using small molecule kinase inhibitors. Treatment of representative murine (PaSC) and human (h-iPSC-PDAC-1) PSC with the Jak1/2 inhibitor ruxolitinib reduced STAT3 phosphorylation and decreased cell proliferation (Fig. 3a,b). The absence of PARP cleavage, as assessed by immunoblot, and retention of cell adherence observed by light microscopy, suggest that these cells did not undergo apoptosis in response to Jak1/2 inhibition. Treatment of cells with the MEK inhibitor MEK162 produced more variable results. For instance, while treatment of murine PaSC cells with MEK162 did not alter cell proliferation, as assessed by MTT and light microscopy (Fig. 3c), there was a modest but statistically significant reduction in proliferation of human PSC (Fig. 3d) in response to MEK162. Notably, a trend toward increased activation of the STAT3 pathway was observed following treatment with MEK162, although this did not reach statistical significance. This compensatory survival mechanism has been previously described in pancreatic and color cancer cell lines^46^. Similarly, increased MAPK pathway activation was seen following treatment with the Jak1/2 inhibitor ruxolitinib in PaSC cells (Fig. 3a).

### Treatment with ruxolitinib, but not MEK162, reduces PSC activation *in vitro*

**Figure 4:**
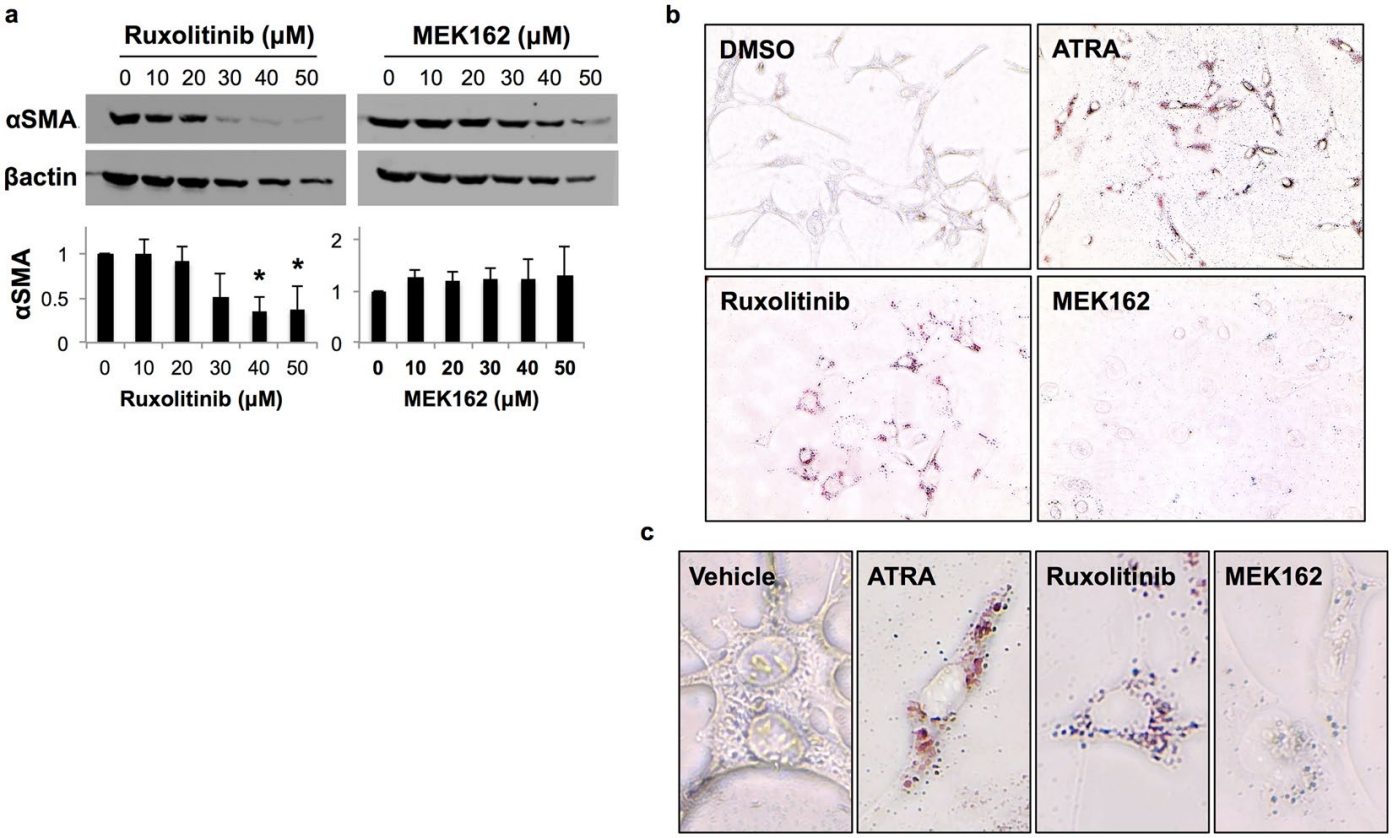
Jak/STAT inhibition reduces PSC activation *in vitro*. (**a**) Lysates of PaSC were taken after 72 hours of treatment with either ruxolitinib or MEK162. α-SMA expression was assessed by immunoblot and quantified by densitometric analysis. Graphs display mean ± SD of 3 biological replicates (* indicates p < 0.05). **(b,c)** PaSC were stained with Oil-Red O to examine quiescence or pseudoquiescence following incubation with vehicle, 10 μM ATRA (positive control), 10 μM ruxolitinib, or 10 μM MEK162 for 48 hours. **(b)** Light microscopy images were taken at 20X magnification. **(c)** Increased magnification of 20X images are shown below at 40X.

Because PSC display reduced cellular proliferation without apparent cell death in response to ruxolitinib, we examined the effect of Jak1/2 or MEK inhibition on biomarkers of cellular activation. By immunoblot, PaSC treated with ruxolitinib exhibited decreased α-SMA expression (Fig. 4a). This trend was not observed in cells treated with MEK162. To confirm these results, PaSC were stained with Oil-Red O, a marker of PSC quiescence or pseudoquiescence, following treatment. Light microscopy results revealed Oil-Red O positive lipid droplets in ATRA (positive control) and ruxolitinib-treated cells. In contrast, untreated and MEK162-treated cells remained Oil-Red O negative (Fig. 4b,c). Taken together, these phenotypic profiles indicate that ruxolitinib treatment reduced PSC activation *in vitro*.

### *In vivo* treatment with ruxolitinib reduces disease severity in a murine model of chronic pancreatitis

**Figure 5:**
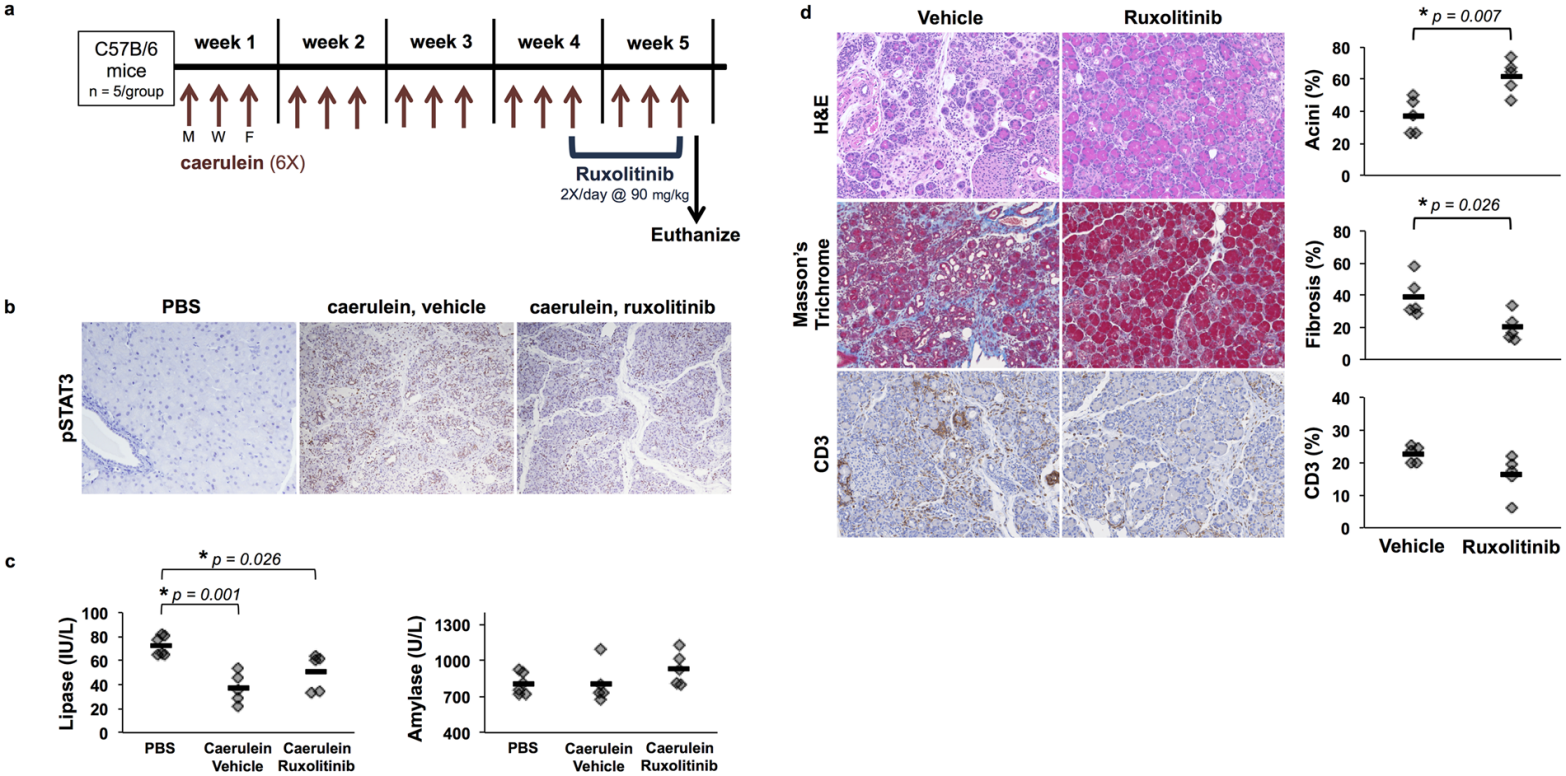
Ruxolitinib reduces disease severity in a murine model of chronic pancreatitis. (**a**) Treatment schedule for *in vivo* experiments. Mice were administered caerulein or PBS (control) via intraperitoneal injection 6X/day, 3 days/week, for 5 weeks. Ruxolitinib was given 2X/day via oral gavage during the final week. **(b)** IHC analysis of pSTAT3 from healthy control mice, pancreatitis-induced mice, and pancreatitis-induced mice treated with ruxolitinib or vehicle control. (**c**) Serum lipase and amylase levels were analyzed upon euthanasia (* indicates p < 0.05). (**d**) FFPE pancreata from caerulein-injected mice treated with either ruxolitinib or vehicle were stained for H&E, Masson’s Trichrome, CD3, and α-SMA. Images are representative of 5 mice per group. Slides were quantified using Vectra automated quantitative pathology imaging system and InForm analysis. Graphs display percent acini, fibrosis, CD3, and α-SMA (* indicates p < 0.05).

To examine the impact of ruxolitinib on biomarkers relevant to CP *in vivo*, we utilized the well-characterized murine model of caerulein-induced chronic pancreatitis (Fig. 5a). In this proof of concept study, oral administration of ruxolitinib for one week in mice with established pancreatitis led to reduced pSTAT3 in the pancreata as determined by IHC (Fig. 5b). This short-term treatment also resulted in a trend toward restored serum lipase levels (Fig. 5c). By IHC analysis, formalin-fixed pancreata showed significantly reduced acinar cell loss and fibrosis. Assessment of CD3^+^ cells by IHC as a biomarker of inflammation also indicated a trend toward a decrease in ruxolitinib-treated mice (Fig. 5d), although no difference in α-SMA staining was observed between groups (data not shown).

## Discussion

We have demonstrated that PSC display constitutive activation of both the Jak/STAT3 and MAPK pathways and secrete an abundance of several immunomodulatory factors, including IL-6 and MCP-1. When treated with the Jak1/2 inhibitor, ruxolitinib, these cells displayed diminished phosphorylation of STAT3 and reduced cell proliferation. This functional phenotype corresponded with a trend toward quiescence or pseudoquiescense, as evidenced by increased Oil-Red O staining and reduced α-SMA positivity. In contrast, treatment with the MEK inhibitor, MEK162, did not alter activation of PSC and had variable effects on cell proliferation. Finally, in a well-characterized *in vivo* murine model of chronic pancreatitis, short-term treatment with ruxolitinib led to preservation of acini and reduced fibrosis by IHC as compared to control animals. These results suggest that disruption of Jak/STAT signaling deserves further investigation as a potential therapeutic strategy in CP. This information is important, considering the lack of any clinically effective strategy to reduce inflammation and fibrosis associated with CP.

In the development of therapeutic strategies for CP, PSC have been a prominent target due to the ability of these cells to promote inflammatory and fibrotic processes during disease. Several studies have shown that PSC-targeted therapies can reduce the severity of CP *in vivo*. Xiao *et al*. demonstrated that, in mice with caerulein-induced CP, treatment with retinoic acid reduced PSC activation and disease severity^47^. Tsang *et al*. showed similar results using an anthraquinone derivative, rhein. In this study, treatment with rhein resulted in reduced PSC activation and fibrosis in caerulein-induced CP^48^. Thus, the available pre-clinical data indicate that strategies to reduce PSC activity may limit the pathological changes associated with CP.

The observed success of ruxolitinib in reducing severity of caerulein-induced CP is promising, as this type of therapy has not been formally evaluated in CP patients to date. Indeed, our results with ruxolitinib are consistent with another pre-clinical study using the AG490 Jak inhibitor. In this report, AG490 reduced secretion of IL-1β by acinar cells and limited inflammatory changes associated with CP in a caerulein-induced rodent model^40^. However, the impact of AG490 on PSC was not evaluated in this prior study.

To date, blockade of pro-inflammatory cytokines and their downstream signaling pathways has not been explored rigorously in human clinical trials of pancreatitis. This may be due to a lack of qualifying pre-clinical data with these agents. Furthermore, preventative or therapeutic clinical trials for CP remain difficult for several reasons, including the heterogeneous nature of the disease and lack of standardized clinical endpoints. Considering these challenges, pre-clinical models remain an important component in advancing treatment options for this disease to ensure that interventions studied in humans have a high likelihood of success.

Although these initial data are promising, it is important to acknowledge some limitations. First, all *in vitro* experiments were conducted in immortalized and non-immortalized cultured PSC. Studies by Gryshchenko *et al*. have demonstrated differences in the threshold of bradykinin-induced Ca^2+^ signaling of cultured PSC compared to freshly isolated PSC within pancreatic lobules^49^. This suggests that differences may exist in the cellular properties of PSC cultured *in vitro* as compared to cells residing in the pancreatic microenvironment. Additionally, interactions between PSC, acinar cells, and immune cells in the pancreas are likely important to PSC biology and are not fully represented by cultured PSC. Accordingly, testing therapeutic concepts in animal models is critical.

Our *in vivo* experiments demonstrated the ability of ruxolitinib to reduce disease severity in caerulein-induced CP. One limitation of this study was the use of a single *in vivo* model. The caerulein-induced murine model is a common and well-characterized model of CP and has been shown to recapitulate several aspects of the pathology present in human CP^50^. However, like any animal model, there are disadvantages to this system. Namely, this model relies upon continuous administration of supramaximal doses of the CCK-analog caerulein, which is distinct from the mechanisms of CP development in human patients and may reduce the clinical relevance of caerulein-induced CP. In mice, caerulein appears to produce a transient up-regulation of STAT3 in the pancreas, not only in PSC, but also within other cells of the parenchyma. For this reason, it is possible that the effects of Jak/STAT inhibition in this model are the result of inhibiting caerulein-dependent events within the pancreas that may or may not be relevant to human disease. However, others have hypothesized that cytokines produced during pancreatic inflammation promote STAT3 signaling within the pancreas which may then cooperate with mutant *KRAS* to drive acinar-to-ductal metaplasia and PanIN formation^39^. This may indicate that inhibition of STAT3 during pancreatic inflammation could limit progression to malignancy, in addition to its potential role in limiting inflammation and fibrosis.

Additionally, our study design examined only short-term dosing with ruxolitinib. Thus, we have not identified the potential long-term effects of this treatment in the context of induced CP. Because the damage caused by caerulein-induced CP is reversible upon cessation of caerulein administration, other clinically-relevant, irreversible animal models should be considered for future studies to assess the impact of long-term inhibition of the Jak/STAT or other pathways in CP^51^. One irreversible model, the recently characterized duct ligation model, mimics the course of human CP caused by pancreatic duct blockage. This or other models may provide additional avenues to evaluate potential therapeutic interventions for CP in the future^52^.

In summary, inhibition of Jak/STAT signaling reduces PSC activation *in vitro* and limits the severity of CP *in vivo*. Thus, this treatment strategy could be adapted for further pre-clinical testing to limit disease progression in CP.

## Methods

### Cell lines and reagents

The PaSC and H-iPSC-CP-1 cell lines were kindly provided by Dr. Hanno Steen^53–55^. The HPF line was purchased from Vitro Biopharma. All other PSC cultures were isolated as described below. All cells were grown in Dulbecco’s Modified Eagle Medium (Gibco, Waltham, MA) with 10% FBS (Gibco) and antibiotics (Gibco). Ruxolitinib (S1378) and MEK162 (S7007) were purchased from Selleck Chemicals (Houston, TX). MTT reagent was purchased from ATCC Bioproducts (Manassas, VA).

### Stellate cell isolation and culture

Human pancreatic stellate cells were obtained in accordance with human subjects research guidelines of The Ohio State University under an Institutional Review Board (IRB)-approved protocol following informed consent and cultured as previously described^24^. Briefly, fresh pancreatic tissue was dissected into 0.5–1 mm^3^ pieces and plated in uncoated culture wells with Dulbecco’s Modified Eagle Medium (Gibco 11965-092) with 10% FBS (Gibco 26140-079) and antibiotics (Gibco 15240-062). These wells were incubated at 37 °C for 2–3 weeks until PSC were observed growing out of the tissue. Following 2 passages, pure PSC cultures were verified via morphology and by α-SMA and GFAP staining. The h-iPSC-PDAC-1 cell line was processed as described above and was then immortalized via transfection with a lentivirus containing SV40 large T-antigen (GenTarget Inc.).

### Microscopic analysis of *in vitro* PSC activation

PSC were plated on chamber slides (Nalgene Nunc International, Rochester, NY) and incubated at 37 °C for 24 hours, after which fresh media containing ruxolitinib, MEK162, or all-trans retinoic acid (R2625, Sigma Aldrich) was added. After 72 hours, slides were stained with Oil-Red O (O0625, Sigma-Aldrich) or α-SMA antibody (ab5694, Abcam). For Oil-Red O staining, cells were fixed with 4% formalin for 30 minutes at room temperature followed by 60% isopropanol for 5 minutes. The Oil-Red O working solution (diluted and filtered according to manufacturer instructions) was added directly to slides for 5 minutes. Wells were rinsed with tap water and slides were mounted with mounting medium (Life Technologies, Carlsbad, CA). Slides were imaged by light microscopy.

For α-SMA staining, wells were fixed with 4% formalin for 20 minutes followed by 0.2% Triton X-100 for 10 minutes. Slides were then blocked with 4% BSA for 60 minutes. Antibody against α-SMA, diluted in 4% BSA was then added to wells and incubated at room temperature for 1 hour. Wells were then mounted using an anti-fade DAPI mounting medium (P36931, Life Technologies). Slides were allowed to cure overnight and then viewed on a fluorescent microscope.

### Analysis of soluble factors in PSC supernatants

Supernatants were collected from PSC cultures at 70–80% confluence and analyzed by bioplex assay (Procarta Cytokine Assay Kit, Affymetrix, Santa Clara, CA). Values were quantified based on a unique standard curve for each analyte.

### Immunoblot and densitometric analysis

Immunoblot was performed as described^56^. Primary antibodies for pSTAT3-Y705 (9145 L) STAT3 (4904 S), pERK (4370 S), ERK (4695 S), PARP (9542 L), and β-actin (4967 S) were purchased from Cell Signaling Technology, Inc. Primary antibody for α-SMA was purchased from Abcam (ab5694). Secondary antibody was purchased from LI-COR (926–32211). Blots were imaged on a LI-COR infrared imager. Densitometry was performed using ImageJ software. All densitometric calculations represent an average of 3 biological replicates.

### MTT assay

MTT assays were performed as recommended by the manufacturer. Briefly, PaSC and h-iPSC-PDAC-1 cells cultures were grown in 96 well plates and treated with ruxolitinib, MEK162, or vehicle control. After 72 hours, MTT reagent (ATCC) was added for 2 hours at 37 °C. Absorbance was measured by a plate reader at 450 nM.

### Caerulein-induced murine model of pancreatitis

All animal studies were conducted in accordance with the guidelines set forth by The Ohio State University Institutional Animal Care and Use Committee (IACUC) under a protocol approved by the Ohio State University Institutional Review Board. In 4–6 week old female C57BL/6 mice, caerulein was administered at 50 μg/kg by intraperitoneal injection. Six hourly injections were performed three days a week for five weeks^57^. During the final week of injections, ruxolitinib was administered twice daily at 90 mg/kg by oral gavage. Mice were sacrificed and blood and pancreata were collected for analysis. Serum levels of amylase and lipase were detected by spectrophotometric analysis using the Alfa Wassermann VetAce analyzer (West Caldwell NJ). Pancreata were formalin-fixed and paraffin-embedded before being stained for H&E (Leica 560 MX, Wetzlar, Germany), CD3 (Aligent Technologies, Santa Clara, CA), and Masson’s Trichrome (Polyscientific Inc., Bay Shore, NY).

### Quantification of histological acinar loss, fibrosis, and inflammation

All analyses of histological specimens were performed using the Vectra and InForm analysis systems (PerkinElmer). For acinar cell loss, 10 images per mouse were taken from Masson’s Trichrome stained slides. InForm was used to train tissue segmentation to calculate the percent of each image that contained acini. Areas of fibrosis or edema were not included. For fibrosis, the same images were used and the tissue segmenter trained to recognize only areas of fibrosis (blue) and calculate the percentage of each image represented by this tissue category. For inflammation, slides stained with anti-CD3 antibody were quantified by first detecting all nuclei in the field and then calculating the percent of nuclei stained with DAB (CD3^+^). For all analyses, the scores from each of 10 photos per slide were averaged to create an average per pancreas. These values, along with the overall average per treatment group, were graphed.

### Statistics

For immunoblot densitometric analysis, values were normalized to the loading control (β-actin) and the no-treatment control for each experiment. Statistical significance for densitometric analysis, MTT, and serum amylase and lipase was determined using a one-way ANOVA with post-hoc Tukey HSD analysis (p < 0.05). Quantification of IHC, including percentage of acini, fibrosis, and CD3 in pancreata, was analyzed by a simple T-test (p < 0.05).
